# Synthesis, characterization, and photocatalytic study of Bi-doped CuS nanoparticles

**DOI:** 10.1039/d5ra03870g

**Published:** 2025-07-14

**Authors:** Asma'a Ahmed Al-Adhreai, A. M. Abdulwahab, A. H. Al-Hammadi, Arwa Al-Adhreai, Zabn Allah M. Alaizeri, Faisal Katib Alanazi, Aeshah Salem, Mohammed ALSaeedy

**Affiliations:** a Physics Department, Faculty of Science, Sana'a University Sana'a Yemen Asma.AlAdhreai@su.edu.ye; b Physics Department, Faculty of Applied Science, Thamar University Dhamar 87246 Yemen abduhabdulwahab@yahoo.com; c Chemistry Department, Faculty of Applied Science, Thamar University Dhamar 87246 Yemen; d Department of Physics and Astronomy, College of Science, King Saud University Riyadh 11451 Saudi Arabia; e Department of Physics, College of Science, Northern Border University Arar 73222 Saudi Arabia; f Department of Physics, Faculty of Science, Taibah University Yanbu 46423 Saudi Arabia; g Department of Chemistry, Maulana Azad of Arts, Science and Commerce Aurangabad 431004 India; h Department of Chemistry "Giacomo Ciamician", University of Bologna 40126 Bologna BO Italy

## Abstract

Pure CuS and Bi-doped CuS nanoparticles (NPs) with varying concentrations (2.5, 5, 7.5, and 10 wt%) of Bi were synthesized using a facile chemical co-precipitation method. Various characterization techniques, including spectroscopy, microscopy, and diffraction analysis, were used to study the prepared samples. XRD confirmed the formation of a hexagonal CuS phase, and Scherrer calculations revealed that crystallite size increased from 15.15 nm (undoped) to 16.22 nm (10% Bi-doped). HRTEM images indicated mixed shapes with an increase in the size of CuS nanostructures due to the incorporation of Bi dopant atoms (16.27–17.16 nm). The presence of dopants was determined using XRF. UV-vis diffuse reflectance spectroscopy (DRS) indicated a band gap narrowing from 1.38 eV (pure CuS) to 1.23 eV (10% Bi-doped), enhancing visible-light absorption. The low band gap of CuS NPs in the current work indicates their significance for use in optoelectronic devices. The absorption coefficient, extinction coefficient, and refractive index have been widely studied. Photoluminescence (PL) spectra showed suppressed recombination in doped samples. Under direct sunlight irradiation, methylene blue (MB) dye was photocatalytically degraded using pure CuS and Bi-doped CuS NPs. The 10% Bi-doped CuS NPs exhibited the highest degradation efficiency (75.77%) and a pseudo-first-order rate constant (*k* = 0.006 min^−1^), attributed to improved charge separation and band structure modulation. These findings highlight Bi-doped CuS as a promising candidate for cost-effective, sunlight-driven environmental remediation technologies.

## Abstract 2

Pure CuS and Bi-doped CuS nanoparticles (NPs) with varying concentrations (2.5, 5, 7.5, and 10 wt%) of Bi were synthesized using a facile chemical co-precipitation method.

## Graphical abstract

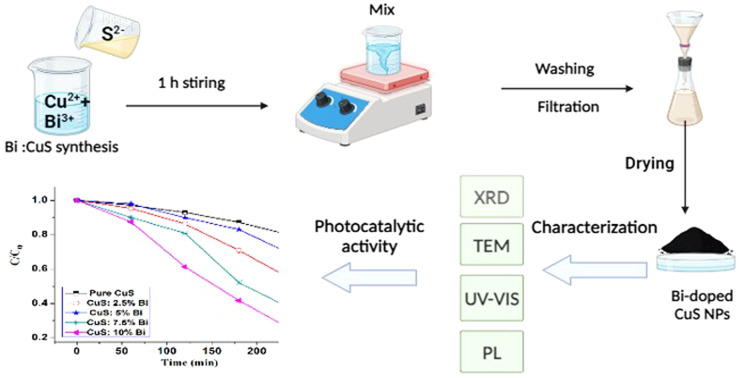

## 1. Introduction

Transition metal sulfides (TMS) like CuS, CoS, and NiS, for example, are currently attracting the interest of researchers because of their earth abundance, excellent environmental compatibility, and low cost, in addition to their high conductivity, good electrochemical stability, and high charge capacity. These TMS have distinctive optical, catalytic, and electrical properties.^1^ Copper sulfide (CuS) is a typical example of a transition metal sulfide that is widely used in many fields by the use of photocatalysts, optical filters, solar cells, and sensors;^2^ in medicine for therapy or as drug carriers or disease diagnosis, in protecting the environment through the removal of pollutants, and lithium ion batteries.^3^ Temperature, molar concentration, capping agents, precursor solution composition, surfactants, and other growth factors all affect the structural and optical characteristics of nanostructured copper sulfide.^4^ Co-precipitation is regarded as one of the simplest and most cost-effective processes due to its simple setup and use of fewer chemical precursors, while also allowing for the incorporation of dopants to modify the electrical characteristics.^5^ For a variety of uses, especially in optoelectronics, photocatalytic activity, and solar energy devices, doping CuS is an interesting way to change the band gap, charge separation, and transport behavior, among other characteristics.^6^ The researchers also reported the incorporation of different doping elements into CuS nanostructures using many techniques to explore their optical, surface morphological, structural, electrical, and magnetic properties. Table 1 summarizes key studies from the last years, highlighting the doping elements, synthesis methods, and main findings. But, to the best of our knowledge, few reports of Bi-doped CuS nanoparticles by the co-precipitation technique have been published.

**Table 1:** The summary of the key findings from several doped CuS studies

Doping element	Synthesis method	Key findings	
Mn	Wet chemical	The band gap increased from 1.6 to 1.7 eV. Increased thermal stability and improved PL intensity	13
Co and Ho	Surfactant-free hydrothermal	A red shift in the bandgap (2.1, 2.0, and 2.0 eV) for pure, Co, and Ho-doped samples respectively. Enhanced photocatalytic properties	14
Fe	Co-precipitation (EDTA-assisted)	Red shift in the bandgap (2.05 eV to 1.97 eV), improving visible light absorption and photocatalytic activity	15
Co	Chemical co-precipitation (EDTA-assisted)	Blue shift in the bandgap (1.96 eV to 2.03 eV). Enhanced photocatalytic properties	16
Ni	Co-precipitation (EDTA-assisted)	The band gap narrows from 2.32 eV (pure) to ∼1.87 eV. Enhanced photocatalytic properties	17
Er	Hydrothermal method	Er doping causes a significant blue shift in the band-gap absorption (from 536 nm to 430 nm). Improved photocatalytic activity	18
La	Microwave irradiation	Decrease in bandgap energy from 4.43 eV to 3.52 eV. Enhancing ion transfer and specific capacitance	19
In	Sonochemical	Optical band gap changes from ∼2.22 eV (undoped) to 1.99–2.15 eV. Enhanced photocatalytic properties	20
Ag	Hydrothermal	Pure CuS shows an absorption peak at ∼953 nm; Ag doping induces a red shift and improved photocatalytic activity	21
Ba	Chemical co-precipitation	The band gap increased from 1.38 eV to 1.82 eV. Enhanced charge carrier separation and stability	22

Bismuth is an excellent contender for improving magnetic, electrical, optical, and photocatalytic properties (Bi: CdZnS, Bi: CdS, ZnBiS, Bi_2_O_3_, BiOCl, BiVO_4_, Bi_2_WO_6_, Bi_2_MoO_6_ and Bi: TiO_2_).^7–9^ Narrow-band gap semiconductor CuS nanocrystals exhibit strong absorption from the visible to the near-infrared spectrum.^10^ Because CuS NPs are inexpensive, safe, and stable in ambient circumstances, they would be perfect for use in clean technology. Recently, there have been attempts to create CuS NPs with high photocatalytic activity so they can be used to resolve environmental problems.^11^ Also, CuS can be utilized as a photocatalyst or adsorbent to remove pollutants from water because it is highly insoluble in it, with a solubility product constant (*K*_sp_) of 1.27 × 10^−36^.^12^

The thermal stability of copper sulfide (CuS) is a critical factor influencing its performance in high-temperature applications, such as solar cells, gas sensors, and thermoelectric devices. Previous studies have shown that CuS undergoes a series of phase transformations when exposed to elevated temperatures. For instance, CuS typically decomposes into non-stoichiometric copper sulfides (Cu_1.8_S and Cu_2_S) around 200–300 °C, accompanied by the release of sulfur dioxide (SO_2_) as the temperature increases to 400–600 °C, these sulfides further oxidize to form copper sulfate (CuSO_4_) and copper oxysulfate (CuO–CuSO_4_). At temperatures above 700 °C, CuSO_4_ and CuO–CuSO_4_ decompose into copper oxide (CuO), with significant weight loss due to the release of SO_2_.^3,23–25^ While TGA studies were not conducted in this work, the expected thermal behavior of CuS is well-supported by existing literature, which provides a foundation for understanding its thermal stability in practical applications.

Semiconductor photocatalysis technology has drawn a lot of attention recently due to its usage in water splitting and the removal of chemical impurities. It offers a comparatively straightforward approach for the chemical conversion of solar energy.^11^ A class of organic chemicals, dyes are used extensively in the printing, culinary, and textile industries. The ecosystem is significantly harmed by dye effluents, the majority of which are extremely toxic and nonbiodegradable. Methylene blue (MB), a phenothiazine derivative, is used to dye fabrics. MB dye was chosen as a model dye because it is extremely harmful and carcinogenic.^26,27^ Additionally, its stable molecular structure and strong visible-light absorption make it ideal for evaluating photocatalytic efficiency.^28^ MB serves as a benchmark in photocatalytic research, enabling direct comparison with other studies.^29^

TiO_2_, a widely available photocatalyst material, is used in the majority of businesses worldwide. Wurtzite-like ZnO is the second most often used photocatalyst for eliminating toxic colors from textile effluents. However, since the sun's rays only create 5% of UV radiation and 46% of visible light, photocatalysis confronts many challenges, one of which is (i) inadequate use of visible light. (ii) Separation is required following activity because the photocatalyst is usually in powder form. TiO_2_ and ZnO have two primary problems that have prevented them from being widely used. As the bandgap increases (*e.g.*, 3.15 eV for TiO_2_ and 3.37 eV for ZnO, respectively), less energy is absorbed.^30^

The photocatalytic efficacy of CuS nanocrystals is, however, restricted by the photogenerated electron pairs' quick recombination.^31^ As is well known, doping semiconductor nanomaterials can efficiently increase charge separation and limit electron–hole recombination, thus increasing photocatalytic efficiency.^21^

While doping CuS nanoparticles with elements such as Fe, Ni, and Mn has been extensively studied, the incorporation of Bi as a dopant remains relatively unexplored. Bismuth (Bi) offers unique properties that make it particularly effective as a dopant in CuS nanostructures. Its relatively large ionic radius (∼1.03 Å) compared to Cu^2+^ induces local lattice distortion when incorporated into the CuS lattice, creating defect states that enhance charge carrier separation. The 6s^2^ lone pair and 6p orbital of Bi^3+^ hybridize with S 3p orbitals, leading to the upshift of the valence band and narrowing of the band gap, thereby enhancing visible-light absorption. Additionally, Bi doping introduces shallow trap states, prolongs charge carrier lifetimes, and suppresses recombination of photogenerated electron–hole pairs. These effects collectively contribute to improved photocatalytic and optical performance. Furthermore, Bi is low-cost, abundant, and environmentally friendly, making it a suitable choice for scalable photocatalytic and optoelectronic applications.^32,33^

In this work, we demonstrate that Bi doping reduces the band gap from 1.38 eV (undoped CuS) to 1.23 eV (10% Bi) and enhances photocatalytic degradation efficiency of methylene blue from 20.7% to 75.77%, with the pseudo-first-order rate constant increasing from 0.0024 to 0.006 min^−1^. These improvements result from the combined effects of Bi-induced band structure modulation and enhanced charge carrier separation. This study provides a quantitative assessment of Bi doping effects in CuS and expands current understanding of dopant-enabled performance tuning. Our findings are in agreement with recent advances in defect engineering, band gap manipulation, and visible-light photocatalysis in nanostructured materials.^34–36^

This work systematically investigates the structural, optical, and photocatalytic properties of Bi-doped CuS nanoparticles and provides new insights into how Bi doping can advance the performance of metal sulfide-based semiconductor nanomaterials.

## 2. Experimental techniques and conditions

### 2.1. Materials used

Sodium sulfide hydrate (Na_2_S·*x*H_2_O, HIMEDIA, ≥65%), copper chloride 2-hydrate (CuCl_2_·2H_2_O, Sigma Aldrich, ≥99%), bismuth nitrate pentahydrate (Bi(NO_3_)_3_·5H_2_O, Fluka, ≥98%), and Methylene blue (MB) (C_16_H_18_ClN_3_S·3H_2_O, Sigma-Aldrich) are used as starting materials. No further purification was required for any of the reagents. All stages of the reaction are carried out in an aqueous medium with distilled water.

### 2.2. Synthesis of pure and Bi-doped CuS nanoparticles

Pure and Bi-doped CuS nanoparticles were synthesized using a chemical co-precipitation method. For the metal precursor solution, 2 M copper(ii) chloride dihydrate (CuCl_2_·2H_2_O) and 2 M bismuth nitrate pentahydrate (Bi (NO_3_)_3_·5H_2_O) were each dissolved in 10 mL of distilled water and stirred for 20 minutes at room temperature until a clear green solution formed. The Bi doping concentrations were adjusted to 0, 2.5, 5, 7.5, and 10 wt% relative to copper by varying the amount of Bi precursor added to the Cu solution.

Separately, a 2 M sodium sulfide nonahydrate (Na_2_S·9H_2_O) solution was prepared by dissolving the salt in 25 mL of distilled water, followed by stirring for 20 minutes to ensure complete dissolution.

The Na_2_S solution was then added dropwise to the Cu–Bi solution under continuous magnetic stirring at room temperature (25 °C). The resulting mixture was stirred for 1 hour to facilitate nanoparticle formation. The solution was then left undisturbed and aged at room temperature for an additional 2 hours to promote further crystal growth. No external pH adjustment was applied during synthesis; the reaction proceeded at its natural pH.

The resulting precipitate was separated by filtration, and washed three times each with distilled water and ethanol to remove unreacted ions and surface impurities. The final product was dried in a hot air oven at 100 °C for 3 hours, yielding a dark greenish powder. The dried samples were gently ground in an agate mortar and pestle to obtain a fine powder suitable for structural, optical, and photocatalytic characterizations.

### 2.3. Characterization techniques

Different characterization investigations were used to analyze the produced pure and Bi-doped CuS nanoparticles. By using an XD-2 X-ray diffractometer with CuK-1 radiation of *λ* = 0.154056 nm, the identity and phase of the products were determined. For investigating the optical properties, a UV-VIS-NIR diffuse reflectance spectrophotometer (V-570) was utilized. An FS5 spectrometer (Edinburgh) was used to record the PL spectra. The TEM images were obtained using a transmission electron microscope (TEM) (JEM-2100, Japan), which was used to determine the interior morphology (final shape and size). ImageJ 1.53e software was used for processing and analyzing high-resolution transmission electron microscopy (HR-TEM) images. Also, Origin Pro 8 was used for plotting graphs and analyzing experimental data. A total reflection X-ray fluorescence (TXRF) spectrum from the Bruker S8 TIGER was used for the elemental analysis.

### 2.4. Photocatalytic activity

The photocatalytic activity of pure and Bi-doped CuS nanoparticles was assessed through the degradation of methylene blue (MB) dye under natural sunlight. In each experiment, 10 mg of the catalyst was dispersed in 40 mL of an aqueous MB solution with a concentration of 2 × 10^−5^ M. To achieve adsorption–desorption equilibrium between dye molecules and the catalyst surface, the suspension was stirred in the dark for 30 minutes prior to irradiation. Although the catalyst–dye mixtures were stirred in the dark for 30 minutes to achieve adsorption–desorption equilibrium, the adsorption efficiency during this phase was not separately quantified, as our primary focus was on overall photocatalytic performance under sunlight. This approach follows standard methodology reported in similar studies.^37,38^ Following equilibration, the mixture was exposed to direct sunlight between 10:00 a.m. and 2:00 p.m. on clear-sky days in June 2023 in Dhamar, Yemen. The average solar intensity during irradiation was approximately 125 000 lux ± 10 000, as measured using a digital lux meter, consistent with values reported in the literature.^39^ The reaction was conducted at ambient pressure and temperature (27–28 °C) under continuous stirring. Throughout the 4 hour irradiation period, 5 mL aliquots were withdrawn at regular intervals using a syringe to monitor degradation progress. Each aliquot was centrifuged at 6000 rpm for 10 minutes to remove suspended photocatalyst particles. The absorbance of the clear supernatant was measured using a UV-vis spectrophotometer at 664 nm, corresponding to the maximum absorbance of MB.

## 3. Result and discussion

### 3.1. XRD analysis

**Fig. 1:**
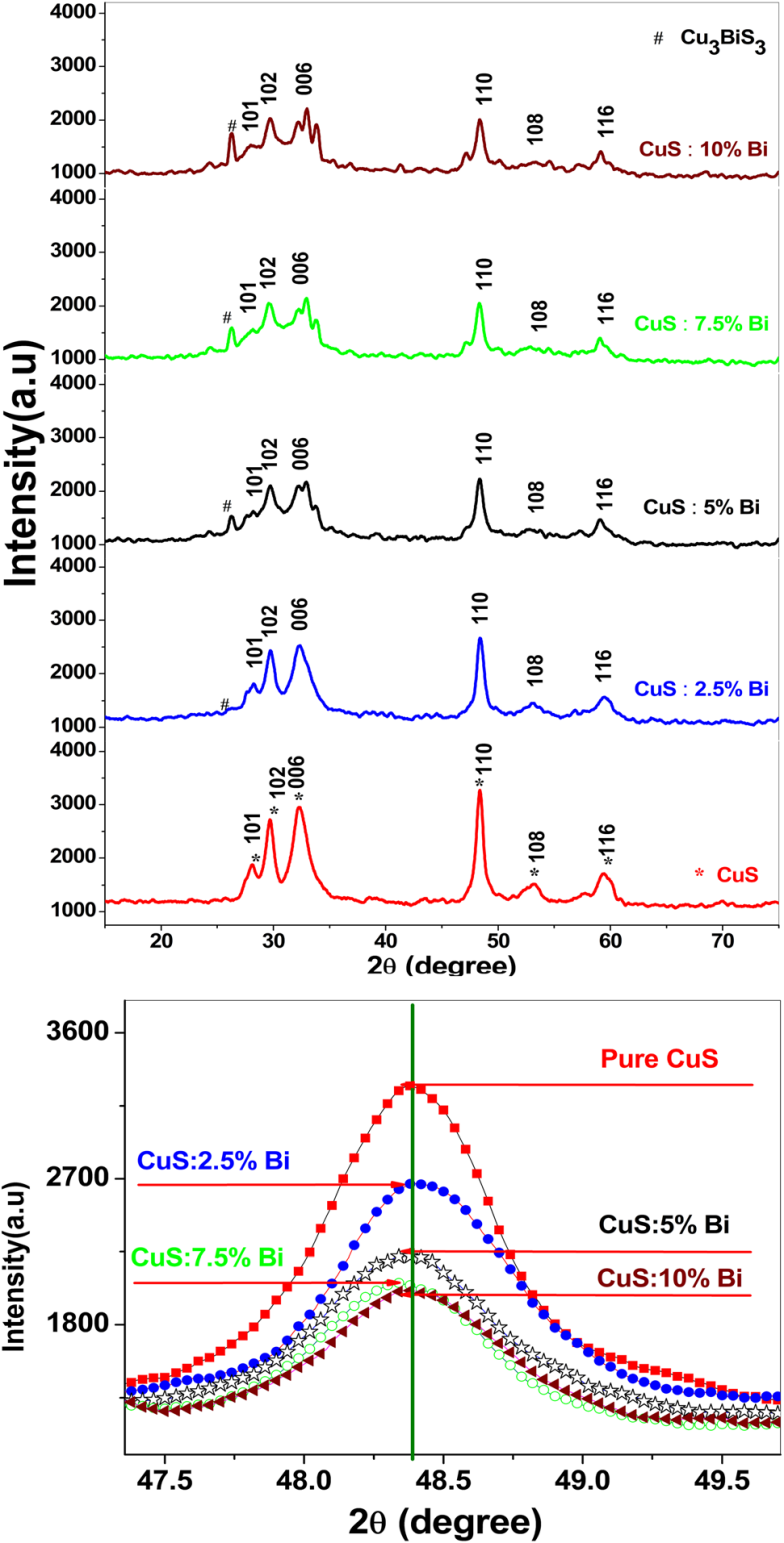
(a) XRD spectra of pure and Bi-doped CuS nanoparticles. The diffraction peaks correspond to the hexagonal phase of CuS (JCPDS no. 06-0464). The peaks are labeled with their respective Miller indices (*hkl*). A slight shift in the peaks is observed with increasing Bi doping concentration, indicating lattice contraction due to the incorporation of Bi^3+^ ions. The spectra demonstrate the successful formation of the CuS phase and the influence of Bi doping on the crystal structure. (b) XRD peak shifting of pure and Bi-doped CuS nanoparticles. The observed peak shifts to higher 2*θ* values with increasing Bi doping concentration, indicating lattice contraction due to the incorporation of smaller Bi^3+^ ions into the CuS lattice. These shifts suggest changes in the lattice parameters and strain within the crystal structure, which are consistent with the observed variations in crystallite size and dislocation density (Table 2). The peak shifting highlights the influence of Bi doping on the structural properties of CuS nanoparticles.

The physical characteristics and degree of crystallinity or amorphous nature are revealed by the XRD patterns.^40^ The XRD patterns of samples produced at various amounts of Bi-doping are compared in Fig. 1a. The CuS hexagonal phase (JCPDS card no. 06-0464, space group *P*6_3_/*mmc*) is clearly visible in the diffraction peaks for all samples. As the bismuth concentration in the CuS sample increases, the XRD peaks become less intense. This implies that as the doping level in the source material increases, the crystallinity of doped CuS NPs decreases. As compared to undoped CuS, the decreased crystallinity of doped samples suggests an increase in disorder brought on by the addition of impurity ions.^41^ The XRD signal shows an additional peak at 2*θ* = 26.18° because of Bi doping. This angle was matched with the JCPDS card no. 27-0149 for Cu_3_Bi_3_S_7_. Fig. 1b displays the diffraction peaks (110) for the produced samples. Compared to the undoped-CuS NPs, the peak location is slightly right-shifted, especially at high dopant concentration values, owing to the greater radius of Bi^3+^ (1.03 Å) compared to Cu^2+^ (0.73 Å). This finding suggests that a small number of Bi^3+^ ions were integrated into the CuS lattice by replacing Cu^2+^ with Bi ^3+^. Bi^3+^ incorporation into the CuS lattice seems to be difficult due to the radius of Bi^3+^ being significantly higher than that of Cu^2+^, possibly limiting the doping level of Bi^3+^ within the CuS lattice.^42^

The Debye–Scherrer formula was used to calculate the crystallite size from the broadening of the (110) diffraction peak:^43^1
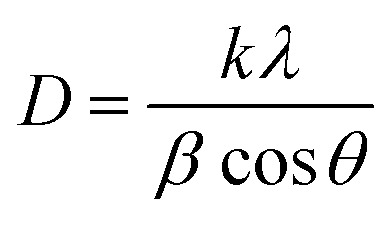
where *k* is a constant given as 0.9, *λ* is the wavelength of X-ray radiation (*λ* = 1.54056), *θ* is the Bragg's angle in degrees, and *β* is the full-width at half-maximum (FWHM) in radians (Table 2).

**Table 2:** XRD data of pure and Bi-doped CuS nanoparticles

Samples	*d* (Å)	2*θ* (degree)	*β* (degree)	*D* (nm)	*a* (Å)	*c* (Å)	*δ* × 10^15^ (m^−2^) dislocation density	*V*_cell_ (Å)^3^
Pure	1.8776	48.38	0.575	15.14701	3.7552	16.3698	4.36	199.91259
2.5% Bi	1.8762	48.44	0.63	13.82574	3.7524	16.2738	5.23	198.44395
5% Bi	1.8784	48.4	0.573	15.19929	3.7568	16.3506	4.33	199.84831
7.5% Bi	1.8791	48.4	0.57	15.27868	3.7582	16.3494	4.28	199.98261
10% Bi	1.8784	48.42	0.537	16.21823	3.7568	16.3404	3.80	199.72364

The following equation is used to calculate the lattice parameters for crystals of pure CuS and those doped with bismuth that have a hexagonal structure:^44^2
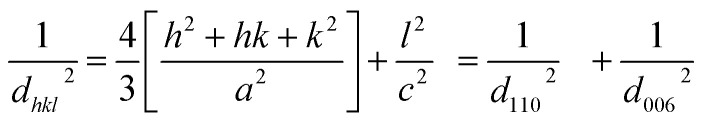
3
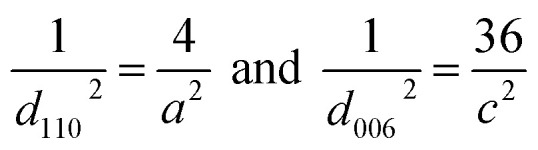
where *d*_*hkl*_ is the interplanar spacing, *h*, *k*, and *l* are the Miller indices, *a* and *c* are the lattice constants. The crystalline peak of the (110) plane is used to calculate lattice parameters (Table 2).

The dislocation density due to crystal imperfections was also determined using:^45^4
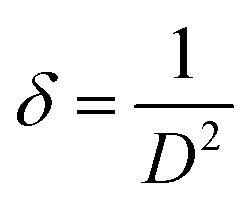


The volume of the unit cell for the hexagonal system is determined by the following equation:^46^5
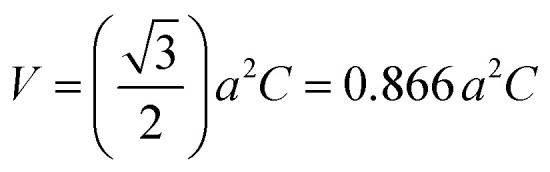


Calculated dislocation density and volume of the unit cell are tabulated in Table 2.

Table 2 summarizes the crystal size, lattice parameters *a* and *c*, dislocation density, and volume of the unit cell. The XRD data in Table 1 reveal several trends in the structural properties of pure and Bi-doped CuS nanoparticles. With increasing Bi doping concentrations, the crystal size (*D*) increases from 15.15 nm (pure CuS) to 16.22 nm (10% Bi), indicating that Bi doping promotes crystal growth. The 2.5% Bi-doped CuS sample has the lowest crystallite size (13.82574 nm) compared to other samples, as indicated by the XRD data. This can be attributed to defects and dislocations where the dislocation density (*δ*) is higher for the 2.5% Bi sample (5.23 × 10^15^ m^−2^) compared to other samples. Higher dislocation density indicates more defects in the crystal structure, which may inhibit crystal growth and result in smaller crystallite sizes.^47^ The lattice parameters (*a* and *c*) show minor variations, with a slight decrease in ‘*c*’ suggesting lattice contraction due to the incorporation of Bi^3+^ ions. The dislocation density decreases at higher Bi doping levels, reaching its lowest value of 3.80 × 10^15^ m^−2^ for 10% Bi-doped CuS, indicating improved crystallinity. The unit cell volume remains relatively stable, with a slight decrease consistent with the observed lattice contraction. These results highlight the influence of Bi doping on the structural properties of CuS nanoparticles.

### 3.2. TEM analysis

**Fig. 2:**
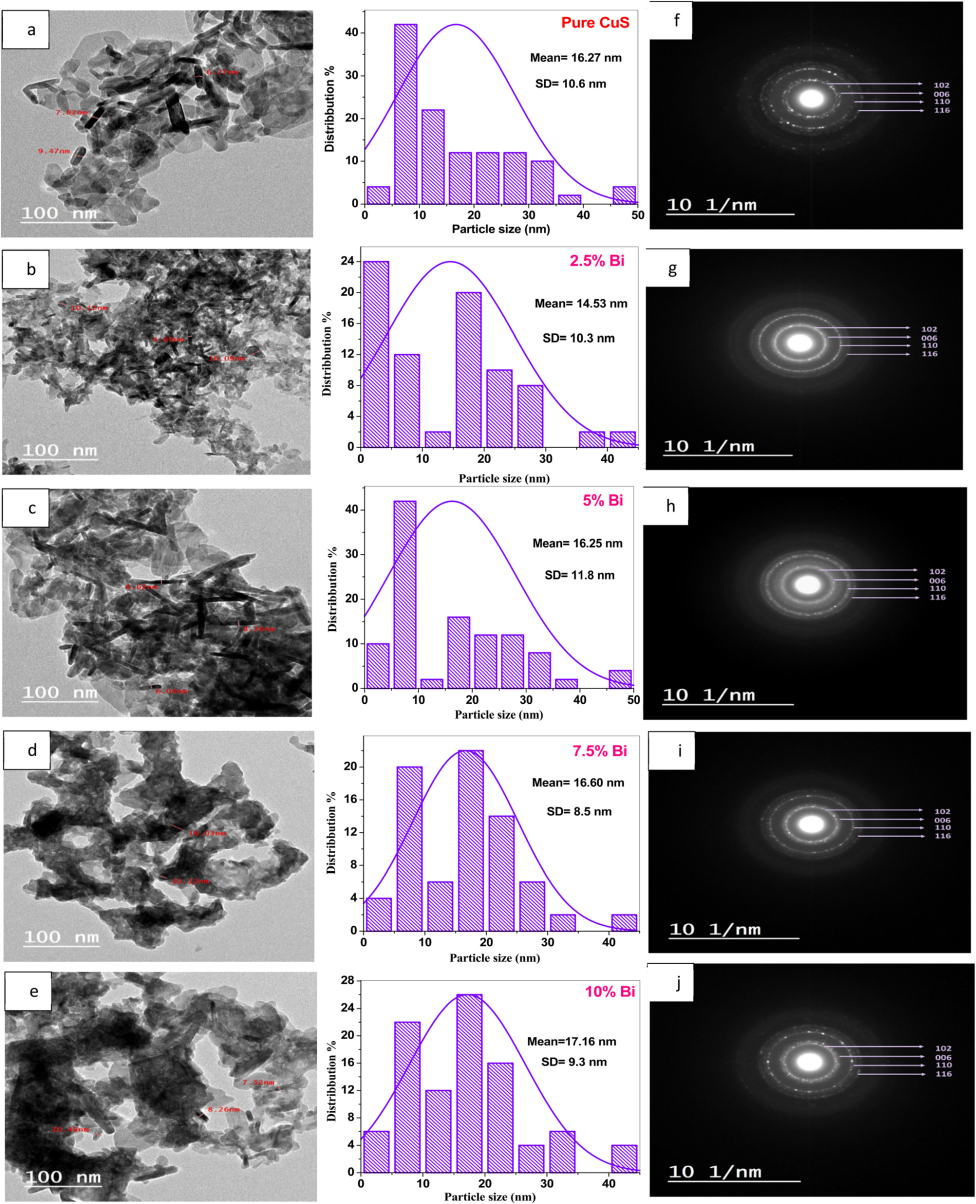
TEM images, particle size distribution histograms, and SAED patterns of Bi-doped CuS nanoparticles. (a–e) TEM images and corresponding particle size distribution histograms for pure CuS and CuS doped with 2.5%, 5%, 7.5%, and 10% Bi. The images reveal the morphological changes and size distribution of nanoparticles with increasing Bi doping. (f–j) SAED patterns for the same samples, showing the crystalline structure and confirming the polycrystalline nature of the nanoparticles.

To gain a deeper comprehension and confirm the samples' microstructure, transmission electron microscopy (TEM) was employed.^48^ TEM images and the particle size distribution histograms of un- and Bi-doped samples at various concentrations are shown in Fig. 2. Images show how the dopant atoms have an impact on the shape of the synthesized samples. The TEM images of the pure CuS in Fig. 2a clearly demonstrate that the prepared nanoparticles have a mixed morphology, with a small percentage of hexagonal-shaped particles dominated by rod-shaped particles with an average size of 16.27 nm. Irregular shapes with a more nanorod shape (14.53 nm) were obtained with 2.5% Bi-doped CuS nanoparticles (Fig. 2b). The TEM image of 5% Bi-doped CuS nanoparticles is displayed in Fig. 2c. A mixture of distinctive rod-like particles and amorphous with an average size of 16.25 nm. With an average size of 16.60 nm, the cluster of nanoparticles in Fig. 2d is highly agglomerated and contains a mix of rectangular, nanorods, and spherical-shaped particles. According to Fig. 2e, 10% Bi-doped CuS exhibits the formation of rod-like nanostructures with particle sizes around 17.16 nm, and the particles exhibit some agglomeration. The shape and size of the CuS nanostructures are typically influenced by doping elements, time, molarity, reaction temperature, and reactive concentration.^20^ An increase in surface energy could be the cause of the aggregation seen in all samples' HRTEM images. Particles with greater surface energies tend to expand more quickly than those with lower surface energies, which results in non-uniform growth. Particles often develop into larger particles in an effort to lower their surface-free energy. Together, the polydispersed particles create a structure that look like a hexagonal rod.^49^

Fig. 2f–j exhibits the corresponding selected area electron diffraction (SAED) pattern for the pure CuS, 2.5, 5, 7.5, and 10% Bi-doped CuS nanoparticles, respectively. By calculating the *d*-spacing with the aid of the ImageJ program, it was possible to determine the planes corresponding to various rings. The diffraction patterns exhibit the lattice planes (102), (006), (110), and (116) of the polycrystalline nature of the hexagonal structure of the CuS particles (JCPDS no. 06-0464), which is in good agreement with the XRD result. Additionally, the close match between the particle sizes observed in HRTEM (16.27–17.16 nm) and the crystallite sizes estimated from XRD (15.15–16.22 nm) suggests that the nanoparticles are predominantly single-crystalline in nature, with minimal aggregation of multiple crystallites within individual particles.^50^

### 3.3. Total reflection X-ray fluorescence

TXRF analysis was used to identify the element and perform a quantitative study to verify the presence of Bi in the experiment-prepared CuS nanoparticle structure. The results of the element content in the samples are shown in Table 3. The primary components in each sample are Cu and S, whereas the minor elements are Bi. The results from TXRF confirm the use of the co-precipitation approach to achieve the proper quantity replacement of the ion Bi^3+^ in the CuS nanoparticle structure. As can be observed, the amount of Bi present measured by TXRF increased with increasing Bi concentrations during synthesis. In addition, increasing the amount of Bi reduces the amount of Cu. Traces of other elements (Cl, P, and Ca) were found.

**Table 3:** TXRF analysis result of CuS and Bi-doped CuS

Samples	Cu, %	S, %	Bi, %	Cl, P, Ca (traces), %
Pure	55.25	44.39	—	0.36
2.5% Bi	53.13	45.04	1.43	0.40
5% Bi	51.78	43.87	3.98	0.37
7.5% Bi	49.37	42.89	7.37	0.37
10% Bi	45.88	42.68	11.06	0.38

### 3.4. Optical properties analyses

**Fig. 3:**
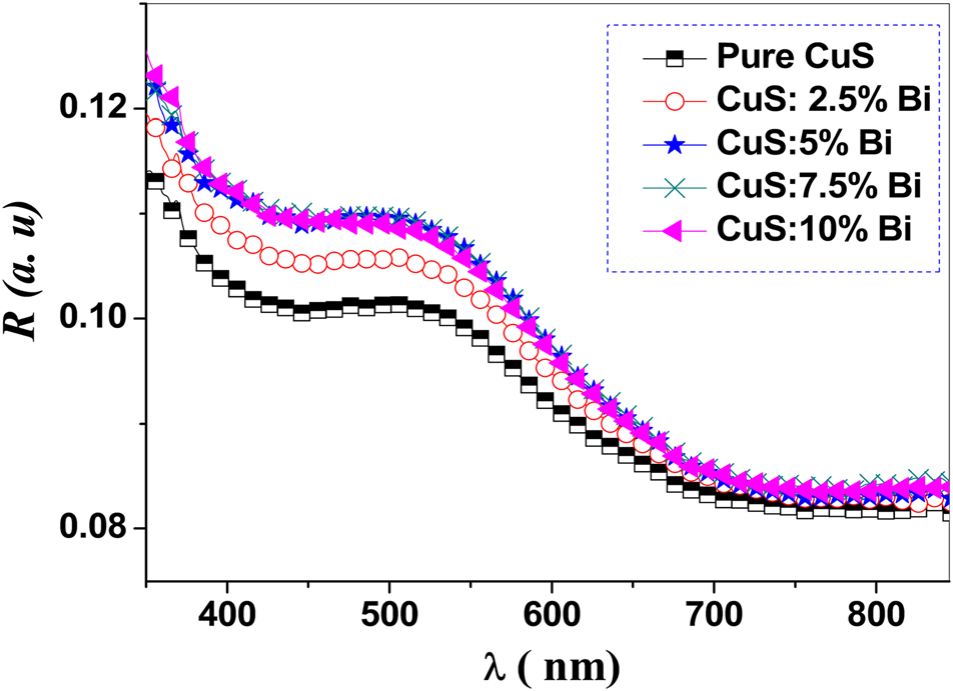
Diffuse reflectance (DR) spectra of pure and Bi-doped CuS nanoparticles. The spectra show the reflectance (%) as a function of wavelength (*λ*) in the range of 350–850 nm. A decrease in reflectance is observed with increasing Bi doping concentration, indicating enhanced light absorption in the visible region. This behavior is attributed to the narrowing of the band gap due to Bi doping, as confirmed by Tauc plot analysis (see Fig. 5). The results demonstrate the potential of Bi-doped CuS nanoparticles for optoelectronic and photocatalytic applications.

The UV-visible diffuse reflectance spectra of CuS doped with Bi were measured in the range of 350–850 nm. It gives helpful information regarding the optical bandgap of the pure CuS and Bi-doped samples. Fig. 3 exhibits the diffuse reflectance of the obtained nanostructures. In the visible range, the prepared samples exhibited a high reflectance spectrum, but in the IR region, they had a low reflectance spectrum.

**Fig. 4:**
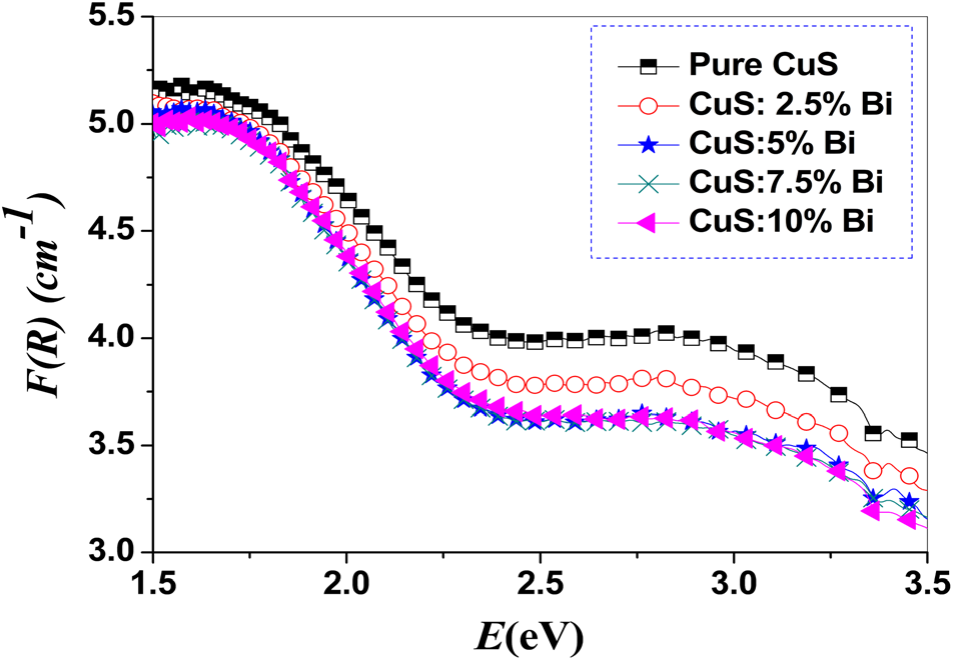
Absorption coefficient of pure and Bi-doped CuS nanoparticles. The graph shows the absorption coefficient as a function of photon energy for pure CuS and CuS doped with varying concentrations of Bi (2.5%, 5%, 7.5%, and 10%). The data demonstrates how the absorption properties of CuS nanoparticles change with increasing Bi doping, which is critical for understanding their photocatalytic or optoelectronic performance. The results indicate that Bi doping enhances the absorption coefficient, suggesting improved light-harvesting capabilities for potential applications in photocatalysis or solar energy conversion.

The reflectance data were converted into absorption data using the Kubelka–Munk equation. This equation is used to calculate the absorption coefficient *F*(*R*) from the reflectance curves and get the band gap energy *E*_g_.^51^6
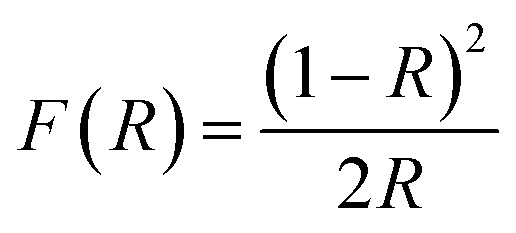
where *F*(*R*) represents the absorption coefficient *α* and *R* refers to reflectance. Fig. 4 shows the obtained *F*(*R*) *versus* photon energy. From Fig. 4, it is obvious that the absorption coefficient decreases as photon energy increases.

The Tauc equation was used to calculate the band gap energy (*E*_g_):^52^

**Fig. 5:**
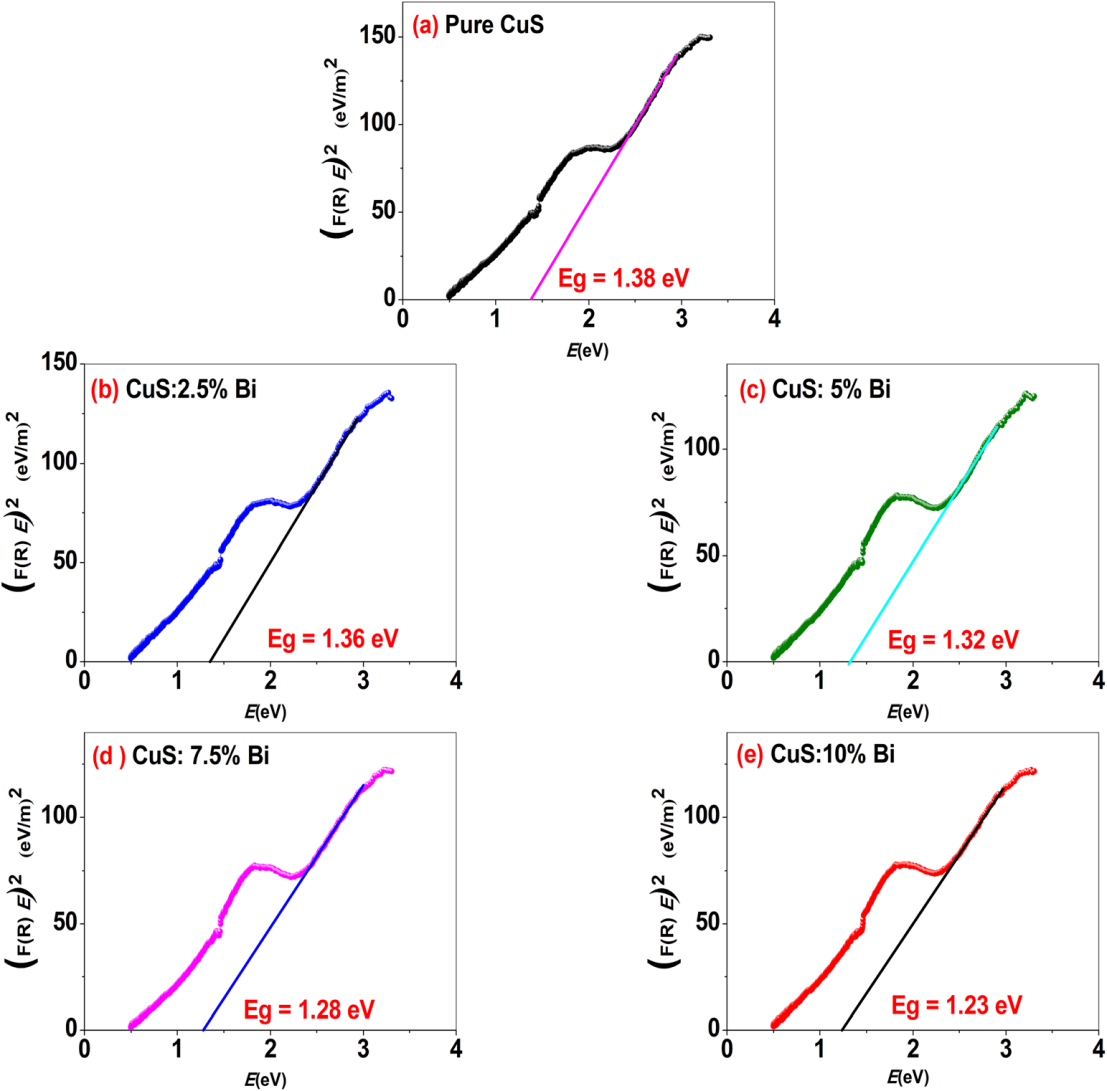
Tauc plots of (*F*(*R*)*E*)^2^*versus* photon energy (*E*) for (a) pure CuS and Bi-doped CuS nanoparticles with increasing Bi concentrations: (b) 2.5%, (c) 5%, (d) 7.5%, and (e) 10%. The plots are used to determine the optical band gap by extrapolating the linear region of the curve to the *x*-axis. A decrease in the band gap is observed with increasing Bi doping concentration, indicating enhanced light absorption in the visible region. These results demonstrate the influence of Bi doping on the optical properties of CuS nanoparticles and their potential for optoelectronic and photocatalytic applications.

7 (*F*(*R*)*E*)^*n*^ = *A*(*E* − *E*_g_)

where *E* represents photon energy (eV), *A* is a constant and *n* is equal to 1/2 for allowed indirect transitions and 2 for allowed direct transitions. CuS is a direct band gap semiconductor, as reported in prior studies.^22,53^ Therefore, we used *n* = 2 in the Tauc equation to determine the direct optical band gap by plotting (*F*(*R*)*E*)^2^*versus* photon energy (*E*). The extrapolation of the linear portion of the Tauc plots intersected with the photon energy axis to produce the band gap energy (*E*_g_). (Fig. 5). The measured band gap values of Cu_1−*x*_Bi_*x*_S (*x* = 0.00, 0.025, 0.05, 0.075, and 0.1) nanoparticles are 1.38, 1.36, 1.32, 1.28 and 1.23 eV correspondingly. It is observed that with increasing Bi content, the estimated optical energy gap *E*_g_ in direct decreases. The lowest *E*_g_ value (1.23 eV) is obtained for 10% Bi-doped concentrations, as seen in Fig. 5. The decrease in optical band gap with increasing Bi^3+^ concentration is attributed to an increase in dopant density and the formation of a continuous density of states, which means a band separating shallow-level acceptor impurities near the VB edge from shallow-level donor impurities near the CB.^54^

The observed increase in crystallite size from 15.15 nm (undoped CuS) to 16.22 nm (10% Bi-doped CuS) can be attributed to the ionic radius mismatch between Bi^3+^ (1.03 Å) and Cu^2+^ (0.73 Å). The substitution of larger Bi ions into the CuS lattice introduces local lattice strain that is relieved during particle growth, promoting fewer nucleation sites and resulting in larger crystallites. This behavior has been reported in similar doped nanostructures, where larger dopant ions facilitate grain coalescence and growth due to enhanced atomic diffusion and reduced surface energy barriers.^55,56^

Bi^3+^ doping also modifies the electronic structure of CuS through hybridization between Bi 6s/6p and S 3p orbitals, which causes an upward shift in the valence band and introduces shallow defect states within the band gap. These states reduce the required energy for electronic transitions, leading to band gap narrowing. Density functional theory (DFT) simulations in doped chalcogenides have shown similar band-edge realignments and redshifts due to dopant-induced orbital overlap and charge redistribution.^55,57^

There are no reports of band gap studies on Bi doped CuS nanoparticles for comparison. Pure CuS nanoparticles have a narrower band gap than bulk covellite CuS, which has a 1.85–2.2 eV characteristic band gap energy.^3^ The narrowing of the band gap can be attributed to the aggregation of small CuS crystallites into the clusters.^49^ In this study, we obtained a band gap of 1.38 eV for pure CuS nanoparticles, which is slightly higher than the 1.3 eV reported by Singh *et al.*^58^ This difference may be attributed to variations in synthesis methods or nanoparticle size, as Singh *et al.* used a sonochemical approach, whereas we employed a chemical co-precipitation method. Our results highlight the sensitivity of the band gap to synthesis conditions and provide new insights into the tunability of CuS nanoparticles for optoelectronic applications. This narrowing of the band gap can be employed to enhance the capture of visible light in solar cell applications.^59^

From reflectance (*R*) data, the refractive index (*n*) can be estimated using the Fresnel equation by the following eqn:^60^8
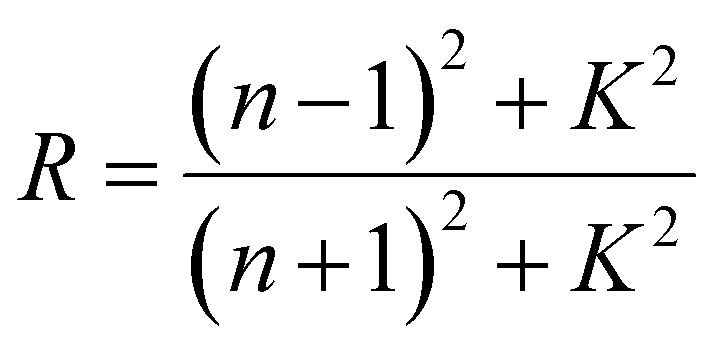
but the extinction coefficient 
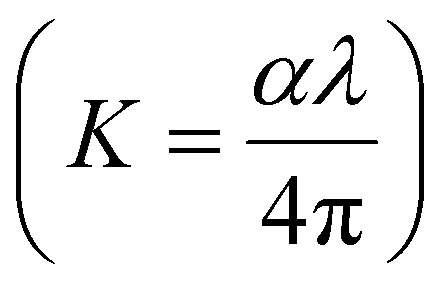
 is very small, then it is neglected; thus, eqn (8) can be rewritten as follows:9
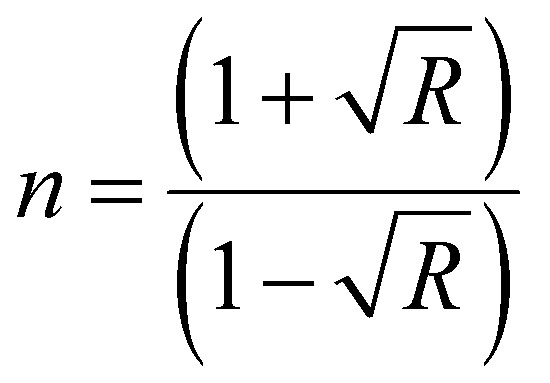


**Fig. 6:**
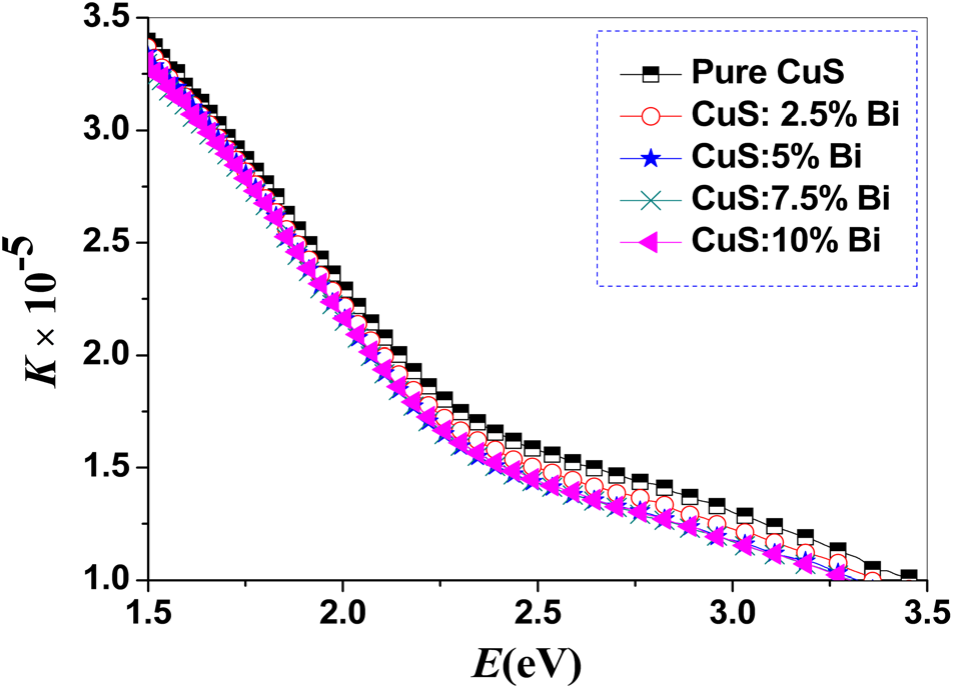
Plots of extinction coefficient (*k*) for pure and Bi-doped CuS nanoparticles. The graph shows the extinction coefficient (*k*) as a function of photon energy (*E*) for pure CuS and CuS doped with varying concentrations of Bi (2.5%, 5%, 7.5%, and 10%). The extinction coefficient, which describes how strongly the nanoparticles absorb light at different wavelengths, increases with higher Bi doping concentrations.

**Fig. 7:**
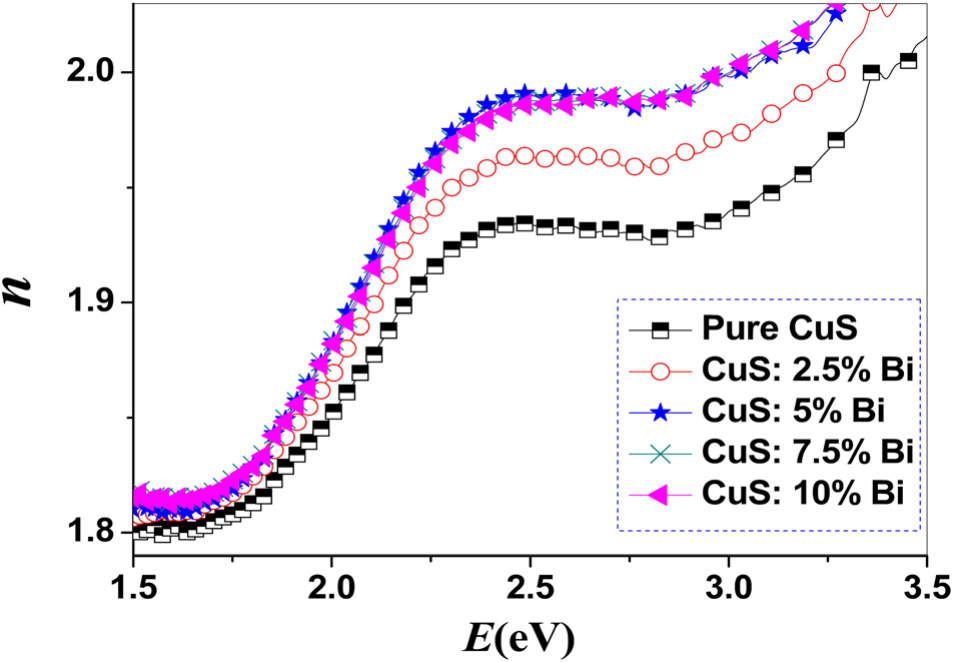
Plots of refractive index (*n*) for pure and Bi-doped CuS nanoparticles. The graph shows the refractive index (*n*) as a function of energy (*E*) for pure CuS and CuS doped with varying concentrations of Bi (2.5%, 5%, 7.5%, and 10%). The refractive index, which describes how light propagates through the material, varies with Bi doping concentration. Higher Bi doping leads to changes in the refractive index, indicating modifications in the optical properties of CuS nanoparticles.

Fig. 6 and 7 show how the extinction coefficient (*K*) and refractive index (*n*) change as a function of incident photon energy (*E*). Fig. 6 demonstrates that when photon energy (*E*) increases, the *K*-values are significantly lowered. Additionally, it is found that as the bismuth concentration in CuS NPs increases, the extinction coefficient for the sample spectrum over the relevant range decreases. According to our results, the refractive index indicates an anomalous dispersion in higher energy zones after a normal dispersion in lower energy zones. Furthermore, Fig. 7 shows that for *E* = 2.44 eV, the refractive index exhibits higher values. Additionally, the refractive index increases as the bismuth concentration increases.

### 3.5. Photoluminescence analysis

**Fig. 8:**
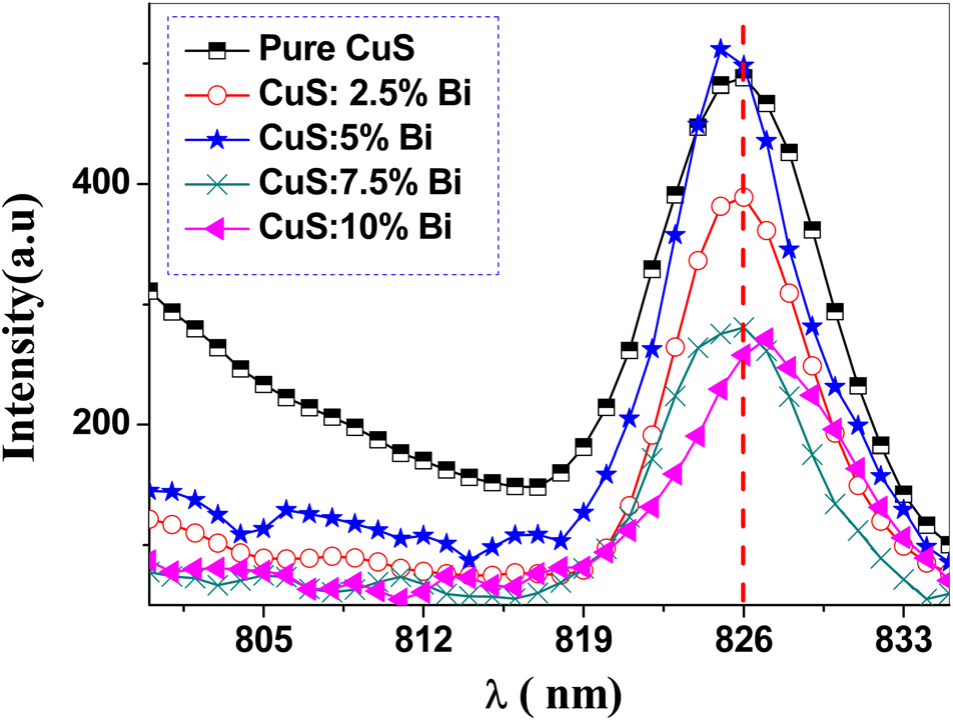
Room-temperature photoluminescence (PL) emission spectra of pure and Bi-doped CuS nanoparticles excited at a wavelength of 535 nm (*λ*_exc_ = 535 nm). The spectra show a strong red emission band centered at 826 nm, which is attributed to the recombination of electron–hole pairs in the CuS lattice. The PL intensity decreases with increasing Bi doping concentration, suggesting that Bi doping reduces the radiative recombination rate.

Fig. 8 displays the PL spectra for undoped and Bi-doped CuS nanoparticles excited at room temperature with a 535 nm wavelength. The band-to-band transition that takes place during the recombination of the electron–hole pairs is clearly related to the strong emission that is centered at 826 nm and is observed in all samples. As seen in Fig. 8, the intensity of PL peaks decreased with increasing Bi dopant concentrations in the doped samples, with the exception of the 5% doped sample. The Bi incorporation mechanism in the nanoparticles helps to explain this unusual intensity variation in Bi-doped CuS nanostructures. There are two methods for adding Bi^3+^ ions to CuS nanostructures: either as interstitials (Bi) or as a replacement for Cu^2+^ ions. The majority of the Bi^3+^ ions may have been substituted into the CuS lattice at doping concentrations of 0.5, 7.5, and 10%; however, at 5% concentration, an excess of Bi^3+^ ions interstitially incorporated into the nanoparticles, resulting in a greater number of lattice defects.^61^ When the concentration of Bi increases to 5 wt%, the intensity of the PL peak increases and shifts towards the lower wavelength side. For 10 wt% of Bi incorporation, the intensity decreases and shifts towards the higher wavelength side. For 2.5 and 7.5 wt% of Bi incorporation, there is no abrupt change in the position of the band. Lower rates of electron and hole recombination are the reason for a decrease in luminescence intensity.

This can be explained by the introduction of non-radiative recombination centers in the CuS lattice due to Bi doping. Bi^3+^ ions may create defects or trap states that promote non-radiative recombination, reducing the number of electron–hole pairs that recombine radiatively.^62^ It is widely known that the photocatalytic activity of semiconductors increases with decreasing PL intensity because it results in a decreased recombination rate for the photogenerated electron–hole pairs.^63^ The progressive decrease in PL intensity with Bi doping is attributed to improved charge carrier separation rather than increased non-radiative recombination. Bi^3+^ ions introduce shallow defect states that capture and extend the lifetime of photogenerated electrons or holes, thereby reducing radiative recombination.^64^ This correlates well with the enhanced photocatalytic activity observed for the 10% Bi-doped CuS sample.

### 3.6. Photocatalytic study

**Fig. 9:**
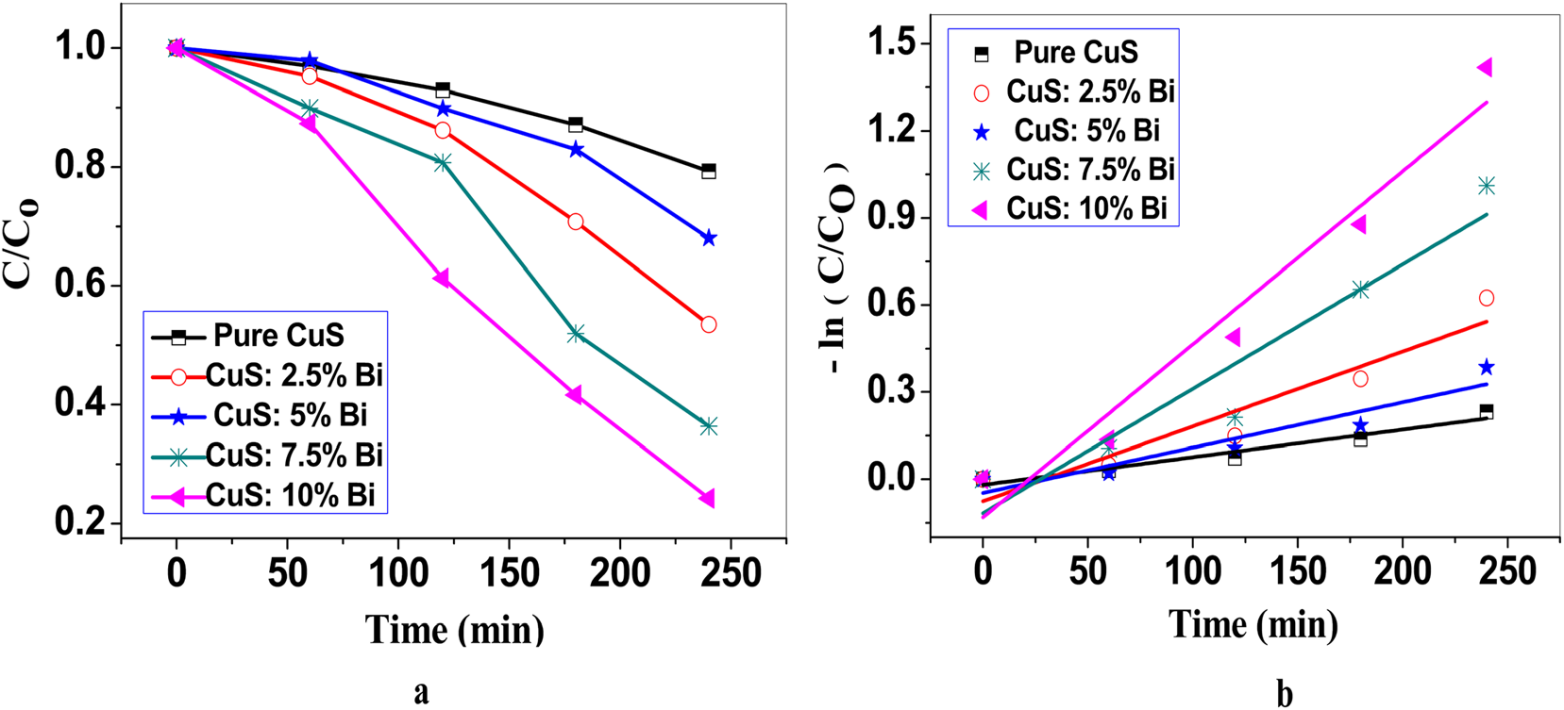
Photocatalytic activity of pure CuS and Bi-doped CuS nanoparticles under sunlight irradiation. (a) Absorbance spectra showing the decrease in MB concentration over time, indicating the photocatalytic activity of the nanoparticles. (b) Kinetic analysis of MB degradation is represented by the plot of −ln(*C*/*C*_0_) *versus* irradiation time, where *C* is the concentration of MB at time *t* and *C*_0_ is the initial concentration. The linear trend confirms pseudo-first-order kinetics, with Bi-doped CuS nanoparticles exhibiting good photocatalytic efficiency compared to pure CuS.

Photocatalytic activity is dependent on electron–hole pair generation, catalyst bandgap, and recombination rate.^65^ By degrading an aqueous solution of the MB dye under direct sunlight irradiation, the photocatalytic activity of the produced catalysts was investigated. To reach equilibrium between adsorption and desorption, photocatalysts were placed in MB solution for 30 min in the dark. After that, mixtures were exposed to direct sunlight. According to the following equation, the degradation efficiency of the samples can be evaluated.^66^10
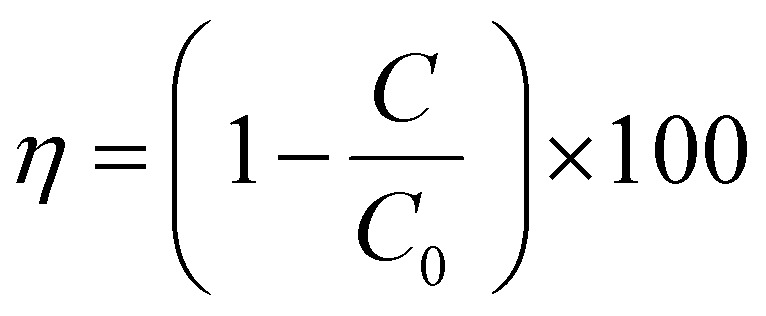
where *C*_0_ is the starting concentration of MB and *C* is the dye concentration after irradiation at a particular time period. The results are shown in Table 4 for this efficiency for all samples. Depending on the MB dye degradation plot (Fig. 9a), the 10% Bi-doped CuS has a greater degradation efficiency (75.77%) when compared to pure and other doped CuS photocatalysts. The degradation efficiency of pure CuS was only 20.70% due to the quick recombination of photogenerated holes and electrons, which also caused the lowest photocatalytic efficiency. With the increase of Bi concentrations from 0.025 to 0.1 for the Bi-doped nanoparticles, photocatalytic efficiency was significantly enhanced.

**Table 4:** Degradation efficiencies, rate constants, and linear regression coefficients for the MB degradation under sunlight in the presence of the synthesized photocatalysts

Sample	% of degradation	Rate constant *k* (min^−1^)	*R* ^2^
Pure	20.70 ± 0.5	0.001 ± 0.0001	0.94
2.5% Bi	46.48 ± 0.7	0.0026 ± 0.0002	0.885
5% Bi	32 ± 1.8	0.0016 ± 0.0001	0.867
7.5% Bi	63.65 ± 2.5	0.0043 ± 0.0003	0.891
10% Bi	75.77 ± 1.9	0.006 ± 0.0004	0.947

Studies show that the photocatalytic degradation of different organic compounds is often studied using the kinetics of decomposition. The Langmuir–Hinshelwood model describes the kinetics of the photocatalytic reaction of organic molecules and is primarily associated with the organic compound's concentration and rate of degradation,^67,68^ which are represented by the equation:^69^11
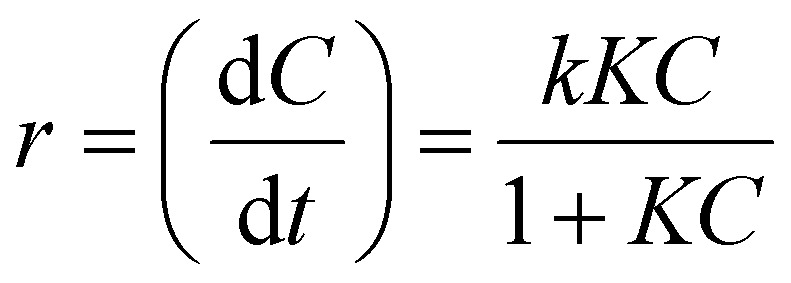
*r* stands for reactant rate (mg L^−1^ min^−1^), *C* for reactant concentration (mg L^−1^), *t* for illumination time, *k* for reaction rate constant (mg L^−1^ min^−1^), and *K* for reactant adsorption coefficient (L mg^−1^). Eqn (11) can be simplified to give the pseudo-first-order kinetic model with a determined rate constant when the absorption is relatively low or the organic composition is low:^70^12
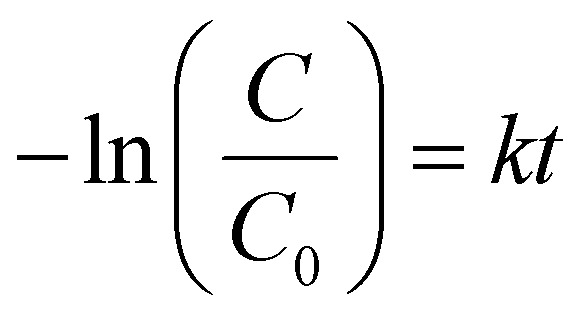
where *k* is the first-order rate constant, *C*_0_ is the initial absorbance, *C* is the absorbance after a time *t*.

Plotting 
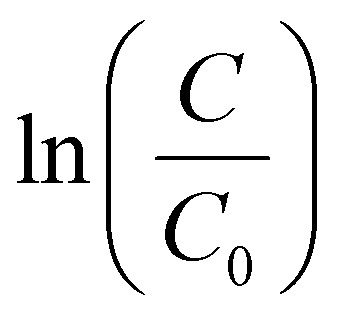
 against time for the MB degrading process with Bi-doped and un-doped CuS photocatalysts is shown in Fig. 9(b). These plots demonstrate that the data is consistently well-fitting and follows eqn (12). The computed values of the pseudo-first order rate constant *k* and *R*^2^ are shown in Table 4. The 10% Bi-doped CuS photocatalyst has a *k* value of 0.006 min^−1^, which is greater than the *k* values for other photocatalysts. This value is approximately 2.5 times greater than that of the undoped CuS (0.0024 min^−1^), demonstrating the substantial improvement in reaction kinetics due to Bi incorporation. This enhancement can be attributed to the narrower band gap (from 1.38 eV to 1.23 eV), which increases visible-light absorption, and the introduction of shallow trap states due to Bi doping, which prolong carrier lifetimes and suppress electron–hole recombination, as evidenced by PL spectra. Also, it is found that the photocatalytic activity of 5% Bi-doped CuS decreased, which might be due to the presence of species on the surface that act as centers for charge carrier recombination.^71^

Under sunlight irradiation, electrons in the valence band are excited to the conduction band, leaving behind holes. These electrons reduce molecular oxygen adsorbed on the catalyst surface to generate superoxide radicals (O_2_˙^−^), which can further react to form hydrogen peroxide (H_2_O_2_) and subsequently produce hydroxyl radicals (˙OH). Meanwhile, holes in the valence band oxidize water or hydroxide ions to generate additional ˙OH radicals. These reactive oxygen species are responsible for the oxidative degradation of MB dye into intermediate and ultimately harmless products. Based on standard CuS photocatalytic pathways, the dominant ROS are expected to be superoxide radicals (O_2_˙^−^) and hydroxyl radicals (˙OH).^65^ Although the generation of superoxide (O_2_˙^−^) and hydroxyl radicals (˙OH) was proposed based on standard photocatalytic mechanisms of CuS-based semiconductors, these reactive species were not experimentally verified in the current work. Future studies will include radical scavenger experiments using isopropanol, benzoquinone, and EDTA to identify the dominant active species involved in MB degradation. The photocatalytic performance of the synthesized Bi-doped CuS nanoparticles was compared with other recently reported materials, as shown in Table 5.

**Table 5:** Comparison of degradation efficiency of Bi-doped CuS nanoparticles with other recently reported photocatalysts

Catalyst	Degradation dye	Conditions	Degradation efficiency	Degradation time (min)	References
CuS	Methylene blue (MB)	Sunlight	90.29%	240	11
CuS	BTB	Sunlight	46.23%	60	72
2.5 mol% Co doped CuS	Methylene blue (MB) (with H_2_O_2_ assistance)	Visible light (40 W, florescent lamb)	99%	20	14
3% Fe doped CuS	Rhodamine B (RhB)	Simulated sunlight (Xe lamp (150 W))	98.53%	80	15
3% Ni doped CuS	Rhodamine B (RhB)	Simulated sunlight (Xe lamp (150 W))	98.46%	60	17
TiO_2_/CuS	Methylene blue (MB)	Xe lamp (300 W)	85.94%	180	73
Cu@CuS	Methylene blue (MB)	Xe lamp (300 W)	90%	200	74
2% Er doped CuS	Methylene blue (MB) (with H_2_O_2_ assistance)	Visible light (40 W florescent lamp)	99%	20	18
3% In doped CuS	Methyl orange (MO)	Simulated sunlight (Xe lamp)	88%	60	20
Cu0.75Ag0.5S	Methylene blue (MB)	Simulated sunlight (Xe lamp (300 W))	93.8%	30	21
CuS	Methylene blue (MB)	Sunlight	20.70%	240	This work
10% Bi doped CuS	Methylene blue (MB)	Sunlight	75.77%	240	This work

As summarized in Table 6, our Bi-doped CuS nanoparticles prepared by the chemical co-precipitation method exhibit a direct band gap narrowing from 1.38 eV (pure CuS) to 1.23 eV (10% Bi), with crystallite sizes increasing slightly from 15.15 to 16.22 nm. This trend is consistent with lattice distortion caused by the larger Bi^3+^ ion substituting Cu^2+^. Compared to other dopants synthesized using more complex or assisted methods (*e.g.*, EDTA-assisted precipitation, hydrothermal, or sonochemical routes), Bi doping demonstrates a competitive balance of structural and optical tuning. The photocatalytic efficiency of 75.77% (under natural sunlight, without additives) achieved by 10% Bi-doped CuS outperforms many reported CuS-based systems, some of which required H_2_O_2_ assistance or high-power Xe lamps. These results highlight Bi doping as a promising strategy to enhance visible-light photocatalysis *via* a simple, low-cost, and scalable route.

**Table 6:** Comparison of band gap, crystallite size, and photocatalytic efficiency of doped CuS nanostructures

Material/dopant	Synthesis method	Band gap (eV)	Crystallite size (nm)	Degradation efficiency (%)	Light source/conditions	Reference
Bi-Doped CuS	Co-precipitation	1.38–1.23	15.15–16.22	75.77	Sunlight/no additives	This work
In-Doped CuS	Sonochemical	2.22–2.15	9.31–8.65	88	Simulated sunlight/MO	20
Fe-Doped CuS	EDTA-assisted co-precipitation	2.05–1.97	6–10	98.53	Xe lamp (150 W)/RhB	15
Ni-Doped CuS	EDTA-assisted co-precipitation	2.01–1.87	5–8	98.46	Xe lamp (150 W)/RhB	17
Co and Ho-doped CuS	Hydrothermal	2.1–2.0	Not reported	99 (with H_2_O_2_)	40 W lamp/MB	14

## 4. Limitations of the study

This study has several limitations that should be acknowledged. First, the range of Bi doping concentrations was limited to 10 wt%, and higher doping levels or co-doping with other elements could provide further insights into the structural and optical properties of CuS nanoparticles. Second, the chemical co-precipitation method used in this study may introduce variability in nanoparticle size and morphology. Alternative synthesis methods, such as hydrothermal or solvothermal approaches, could yield more uniform nanoparticles. Third, the photocatalytic activity was evaluated using only methylene blue (MB) dye under sunlight irradiation. Testing with other organic pollutants or under controlled light conditions could provide a more comprehensive understanding of the photocatalytic performance. Finally, while XRD, HR-TEM, and DRS were used to characterize the nanoparticles, additional techniques such as X-ray photoelectron spectroscopy (XPS) could provide further insights into the chemical states and surface properties of the doped nanoparticles. Future studies could address these limitations to further advance the understanding and application of Bi-doped CuS nanoparticles.

## 5. Conclusion

Un-Doped and Bi-doped CuS NPs were synthesized using the co-precipitation method, and the effect of bismuth doping concentration on the morphological, structural, and optical properties of these materials has been investigated. The hexagonal CuS phase was formed, according to XRD analysis. Because Bi^3+^ ions were successfully incorporated into the CuS lattice, a distinct red shift has been seen in the direct band gap of the Bi-doped NPs. With increasing Bi doping amounts from 0 to 10 wt%, the optical band gap of CuS noticeably narrows. This reduced band gap is particularly advantageous for optoelectronic applications, such as solar cells and photodetectors, where efficient light harvesting in the visible spectrum is critical. Reflection spectra have been used to calculate the values of some important parameters, including the absorption coefficient, extinction coefficient, and refractive index. PL spectra show a strong red emission band at 826 nm at an excitation wavelength of 535 nm. Pure and Bi-doped CuS NPs were used in a photocatalytic process to degrade the dye methylene blue (MB) in the presence of sunlight. The catalyst 10% Bi-doped CuS NPs has the highest photocatalytic degradation efficiency. The enhanced photocatalytic efficiency of 10% Bi-doped CuS (75.77%) suggests its potential for environmental applications, such as the degradation of organic pollutants in wastewater or air purification under sunlight. This study did not directly identify the active radical species responsible for photocatalytic activity. Incorporating radical scavenger analyses in future work will help confirm the mechanistic role of O_2_˙^−^, ˙OH, and h^+^ in the Bi-doped CuS photocatalytic system. These findings highlight the promise of Bi-doped CuS nanoparticles as a cost-effective and efficient material for both optoelectronic devices and photocatalytic systems.

## Consent for publication

All authors agreed to publish the work.

## Author contributions

Asma'a AhmedAL-Adhreai: conceptualization, methodology, software, writing – original draft writing – review & editing. A. M. Abdulwahab: supervision, project administration, data curation, formal analysis, and writing – review. H. Al-Hammadi: supervision, and writing – review, Mohammed ALSaeedy: funding acquisition, resources, Zabn Allah M. Alaizeri: data analysis, Faisal Katib Alanazi: validation, investigation, Aeshah Salem: resources, validation, Arwa Al-Adhreai: visualization, investigation. Mazahar Farooqui: validation, visualization.

## Conflicts of interest

The authors declare no competing interests.

## Data Availability

All data generated or analyzed during this study are included in this published article.
